# Novel mutations of *MYO7A* and *USH1G* in Israeli Arab families with Usher syndrome type 1

**Published:** 2011-12-30

**Authors:** Leah Rizel, Christine Safieh, Stavit A. Shalev, Eedy Mezer, Haneen Jabaly-Habib, Ziva Ben-Neriah, Elena Chervinsky, Daniel Briscoe, Tamar Ben-Yosef

**Affiliations:** 1 The Rappaport Family Institute for Research in the Medical Sciences, Haifa, Israel; 2 The Rappaport Faculty of Medicine, Technion-Israel Institute of Technology, Haifa, Israel; 3 Genetics Institute, Ha’Emek Medical Center, Afula, Israel; 4 Alberto Moscona Department of Ophthalmology, Rambam Medical Center, Haifa, Israel; 5 Department of Ophthalmology, Ha’Emek Medical Center, Afula, Israel; 6 Department of Human Genetics, Hadassah Hebrew University Hospital, Jerusalem, Israel

## Abstract

**Purpose:**

This study investigated the genetic basis for Usher syndrome type 1 (USH1) in four consanguineous Israeli Arab families.

**Methods:**

Haplotype analysis for all known USH1 loci was performed in each family. In families for which haplotype analysis was inconclusive, we performed genome-wide homozygosity mapping using a single nucleotide polymorphism (SNP) array. For mutation analysis, specific primers were used to PCR amplify the coding exons of the *MYO7A*, *USH1C*, and *USH1G* genes including intron-exon boundaries. Mutation screening was performed with direct sequencing.

**Results:**

A combination of haplotype analysis and genome-wide homozygosity mapping indicated linkage to the *USH1B* locus in two families, *USH1C* in one family and *USH1G* in another family. Sequence analysis of the relevant genes (*MYO7A*, *USH1C*, and *USH1G*) led to the identification of pathogenic mutations in all families. Two of the identified mutations are novel (c.1135–1147dup in *MYO7A* and c.206–207insC in *USH1G*).

**Conclusions:**

USH1 is a genetically heterogenous condition. Of the five USH1 genes identified to date, *USH1C* and *USH1G* are the rarest contributors to USH1 etiology worldwide. It is therefore interesting that two of the four Israeli Arab families reported here have mutations in these two genes. This finding further demonstrates the unique genetic structure of the Israeli population in general, and the Israeli Arab population in particular, which due to high rates of consanguinity segregates many rare autosomal recessive genetic conditions.

## Introduction

Usher syndrome (USH) is the most common cause of combined sensorineural deafness and blindness, with a worldwide prevalence of 1 in 16,000 to 1 in 50,000. USH is a recessively-inherited disorder, characterized by the combination of sensorineural hearing loss (SNHL) and retinitis pigmentosa (RP) [1]. RP is a degenerative disease of the retina, characterized by night blindness, followed by gradual restriction of the visual field. Additional ophthalmologic findings include characteristic pigmentation of the midperipheral retina, attenuation of the retinal arterioles, and pale appearance of the optic disc [2]. Based on the severity and progression of hearing loss, age at onset of RP, and the presence or absence of vestibular impairment, the majority of USH cases can be classified into one of three clinical subtypes, the most severe of which is Usher syndrome type 1 (USH1), characterized by profound prelingual hearing loss, vestibular areflexia, and prepubertal onset of RP. Seven USH1 loci (USH1B–USH1H) have been mapped, and the causative genes have been identified for five of them (*MYO7A*, encoding myosin VIIA, *USH1C*, encoding harmonin, *CDH23*, encoding cadherin 23, *PCDH15*, encoding protocadherin 15, and *USH1G*, encoding SANS) [1].

The Israeli population is composed of many ethnic groups, including Jews, Christians, Muslims, Bedouins, Druze, and more. Although several founder mutations underlying different USH types have been reported in the Jewish population [3–5], the genetic basis for USH in the Arab population has not been studied. Among Israeli Arabs, 45% of marriages are between related spouses, half of whom are first cousins. This high rate of consanguinity consequently leads to increased homozygosity of rare mutant alleles in specific families and villages [6]. Homozygosity mapping is an efficient tool for identifying the underlying genetic defects in such families. Here we report four consanguineous Muslim Arab Israeli families segregating USH1. Genetic analysis led to the identification of the underlying mutations in each family, including novel mutations of the *MYO7A* and *USH1G* genes.

## Methods

### Patients

Members of four Muslim Arab consanguineous Israeli families were ascertained for this study. Family TB86 was recruited through Rambam Medical Center in Haifa. Family TB114 was recruited through Hadassah Hebrew University Hospital in Jerusalem. Families TB63 and TB109 were recruited through Ha’emek Medical Center in Afula, Israel. At the time of recruitment all affected individuals met diagnostic criteria for USH1 [1] except individuals II-3 in family TB114 and III-1 in family TB63 who had SNHL only. In total, 22 family members participated in the study (10 affected [seven males, three females] and 11 unaffected [five males, six females]). The study was performed in accordance with the Declaration of Helsinki and written informed consent was obtained from all participants. The research was approved by the local institutional review boards at each of the participating medical centers and by the National Helsinki Committee for Genetic Research in Humans.

### DNA analysis

Venous blood samples were obtained using K3EDTA vacuette tubes (Greiner Bio-One, Kremsmunster, Austria), and genomic DNA was extracted using high salt solution according to a standard protocol [7]. DNA samples were PCR amplified with fluorescent dye-labeled primers flanking microsatellite repeat markers linked to all known USH1 loci (Table 1). PCR products were separated by electrophoresis on an ABI 310 Genetic Analyzer, and genotypes were determined with Genescan and Genotyper software, using a Genescan-400HD-ROX size standard (PE Applied Biosystems, Foster City, CA). Genome-wide homozygosity mapping was performed using the Affymetrix GeneChip Human Mapping 6.0 single nucleotide polymorphism (SNP) array (Affymetrix, Santa Clara, CA). Homozygous regions were calculated using Homozygosity Mapper [8]. For mutation analysis, specific primers were used to PCR amplify the coding exons of *MYO7A*, *USH1C*, and *USH1G*, including intron-exon boundaries. Mutation screening was performed by direct sequencing with the Big Dye Terminator Cycle Sequencing Kit on an ABI 3130xl Genetic Analyzer (PE Applied Biosystems).

**Table 1:** Microsatellite repeat markers linked to all known USH1 loci.

**Locus**	**Chromosome**	**Gene and markers**	**Location (MB)**	**PCR primers for marker amplification**
*USH1B*	11	D11S4179	76	F: GGATGTAAGAGTAACTGGCTCG
				R: GAAAATGTTCTGCCTGAGGG
		D11S1789	76.7	F: ACCAGGAAATTGAGAACCA
				R: TCTGGCCCAACAGAAGT
		D11S4079	76.8	F: CAGCAAGATCCTGTCTCAA
				R: CTCCTTAAAGTGGGGGAGTT
		*MYO7A*	76.8–76.9	
		D11S937	77.5	F: CTAATAAACAAATCCCTCTACCTCC
				R: TAGTCAGTCAGGGACCCAAGT
*USH1C*	11	D11S902	17.4	F: CCCGGCTGTGAATATACTTAATGC
				R: CCCAACAGCAATGGGAAGTT
		*USH1C*	17.5	
		D11S4138	17.7	F: GTGCTGACCGCTCCAAGG
				R: CCAAAGGGGTTAATAGGGGTCCA
*USH1D*	10	D10S537	72	F: CCTACTGTGCCTGGCTAGA
				R: ATTTGGATGAAACCCACG
		D10S606	73	F: TTTGAACCTGGGAGACG
				R: CATGGACATTCTGCTGC
		D10S1694	73.1	F: CCTGTCTGGCCCAGGTA
				R: AGTAGGGGTGCTGCTTGA
		*CDH23*	73.1–73.5	
*USH1E*	21	D21S1437	20.5	F: ATGTACATGTGTCTGGGAAGG
				R: TTCTCTACATATTTACTGCCAACA
		USH1E (locus)	19.8–31.3	
		D21S265	25.8	F: TTAAAGCAATCAATCATGG
				R: GGGTTCTGTGAATATGGG
*USH1F*	10	D10S2537	55.7	F: AAATGGAATATGGGGTTATTAGCA
				R: AATTATCTACATTTCCGCCACTTC
		D10S546	55.7	F: TGTGATGACTAGAAATTCAGTTCA
				R: ACTTCTCAATTACAGGCCCA
		*PCDH15*	55.5–56.5	
		D10S2522	56.3	F: TTTAAACACTGTCCCAACAC
				R: TGAAGGAAAGCCAAACTTA
*USH1G*	17	D17S1301	70.2	F: AAAGAAGATGAAATTGCCATG
				R: TAAAAAGAATGAAGGTAAAAATGTG
		D17S785	71.9	F: ATCCCTGGAGAGTGAAAATG
				R: AAGGCCAACCTGAAAACTAA
		D17S801	72	F: CCTCAAACCGGACAACTATTT
				R: CAGAGAGCAAGATCCTACCTC
		D17S937	72.8	F: CATGGAGGGACTTGCG
				R: TTCCCAGAACCCGGTTT
		*USH1G*	72.9	
*USH1H*	15	USH1H (locus)	65.1–68.4	
		D15S1015	65.6	F: TCACAGAGCGAGACCCTAT
				R: CACAGACTCAGTTTAGACAGAAATC
		D15S131	68.9	F: GAAAGGCACCTCATCTCG
				R: TTAAAAACTCTGGAGCAGCG

## Results

### An *USH1C* mutation in family TB86

**Figure 1:**
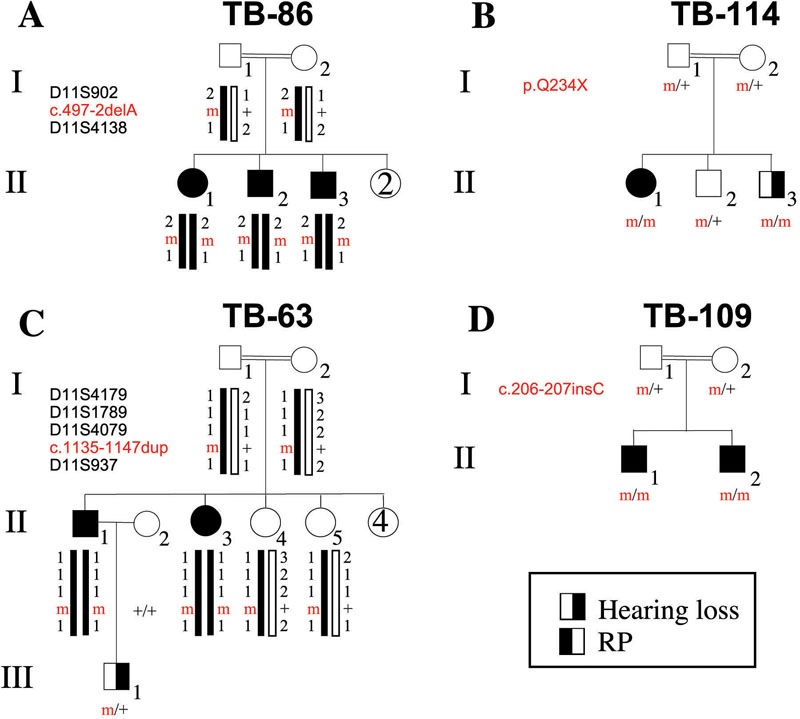
Pedigrees and genotypes of Israeli Arab families with USH1. Shown are four Israeli Arab families segregating USH1. Double lines indicate consanguineous unions. Filled symbols represent affected individuals, whereas clear symbols represent unaffected individuals. Mutation-bearing haplotypes are marked with black bars. **A**: Family TB86. Genetic analysis demonstrates co-segregation of an *USH1C*-linked haplotype and mutation (c.497–2delA) with USH1. **B**: Family TB114. Genetic analysis demonstrates co-segregation of a mutation of *MYO7A* (p.Q234X) with USH1. **C**: Family TB63. Genetic analysis demonstrates co-segregation of an *USH1B*-linked haplotype and a *MYO7A* mutation (c.1135–1147dup) with USH1. **D**: Family TB109. Genetic analysis demonstrates co-segregation of a mutation of *USH1G* (c.206–207insC) with USH1. m, mutant allele; +, wt allele.

Family TB86 is a Muslim Arab family from northern Israel. The parents are first cousins, and three of their five offspring have USH1 (Figure 1A). Bilateral profound SNHL was detected at infancy in all three affected individuals. One manifested a delay in motor development and walked independently at age 2.5, which is consistent with the USH1 phenotype [1]. The other two walked independently at the age of 15 months. Difficulties with night vision were observed at a very young age, and constriction of the visual field (VF) was detected around the age of 10. When we examined the patients, all three were in their 20s. They all had marked VF constriction of 20° to less than 10° and nondetectable electroretinograms (ERG). Funduscopic examination revealed typical retinal bone spicule-type pigment deposits (Table 2).

**Table 2:** Clinical data of individuals with USH1.

**Family**	**Gene and mutation**	**Patient ID and sex**	**Age**	**Vision**						**Hearing**	**Additional findings**
				**Age at diagnosis of vision impairment**	**Visual acuity (best corrected)**	FFERG*		**Visual field**	**Funduscopic findings**		
						**LA (single flash)**	**DA**				
TB-86	USH1C c.497–2delA	II-1 F	30 y	10 y	R 6/15 6/18	NR	NR	R <10° L <10°	Typical retinal bone-spicule pigmentation in mid periphery	Congenital, bilateral profound SNHL	
		II-2 M	26 y	10 y	R 6/90 6/60	NR	NR	R <10° L 10°–20°	Typical retinal bone-spicule pigmentation in mid periphery	Congenital, bilateral profound SNHL	
		II-3 M	25 y	10 y	ND	NR	NR	ND	ND	Congenital, bilateral profound SNHL	
TB-114	MYO7A c.700C>T; p.Q234X	II-1 F	8 y	8 y	ND	ND	ND	ND	Typical retinal bone-spicule pigmentation	Congenital, bilateral profound SNHL	Increased feet tonus and hyperflexia
		II-3 M	8 m	NE†						Congenital, bilateral profound SNHL	
TB-63	MYO7A c.1135–1147dup; p.S383WfsX63	II-1 M	28 y	9 y	ND	ND	ND	ND	Typical retinal bone-spicule pigmentation	Congenital, bilateral profound SNHL	Developmental delay
		II-3 F	11 y	9 y	ND	ND	ND	ND	Typical retinal bone-spicule pigmentation	Congenital, bilateral profound SNHL	
		III-1 M	1 y	NE†						Congenital, bilateral severe SNHL	
TB-109	USH1G c.206–207insC; p.L69PfsX66	II-1	8 y	3 y	R 6/10 6/10	ND	ND	ND	Salt and pepper-like degeneration with mild vascular attenuation, a reduced foveal reflex, and normal optic disks.	Congenital, bilateral severe SNHL	Hypotonia, developmental delay, myopia, astigmatism
		II-2	6 y	5 y	R 6/12 6/12	ND	ND	ND	Salt and pepper-like degeneration with mild vascular attenuation, a reduced foveal reflex, and normal optic disks.	Congenital, bilateral severe SNHL	Situs inversus, myopia, astigmatism

F, Female; M, Male; y, years; m, months; R, Right eye; L, Left eye; NR, Non-Recordable; ND, Not Done; SNHL, sensorineural hearing loss *FFERG, Full Field Electroretinogram; LA, Light Adaptation: Normal amplitude 80–180 μV; Normal latency 30–35 ms; DA, Dark Adaptation: Normal a-wave 100–200 μV; Normal b-wave 400–550 μV †NE, Not further examined regarding RP.

**Figure 2:**
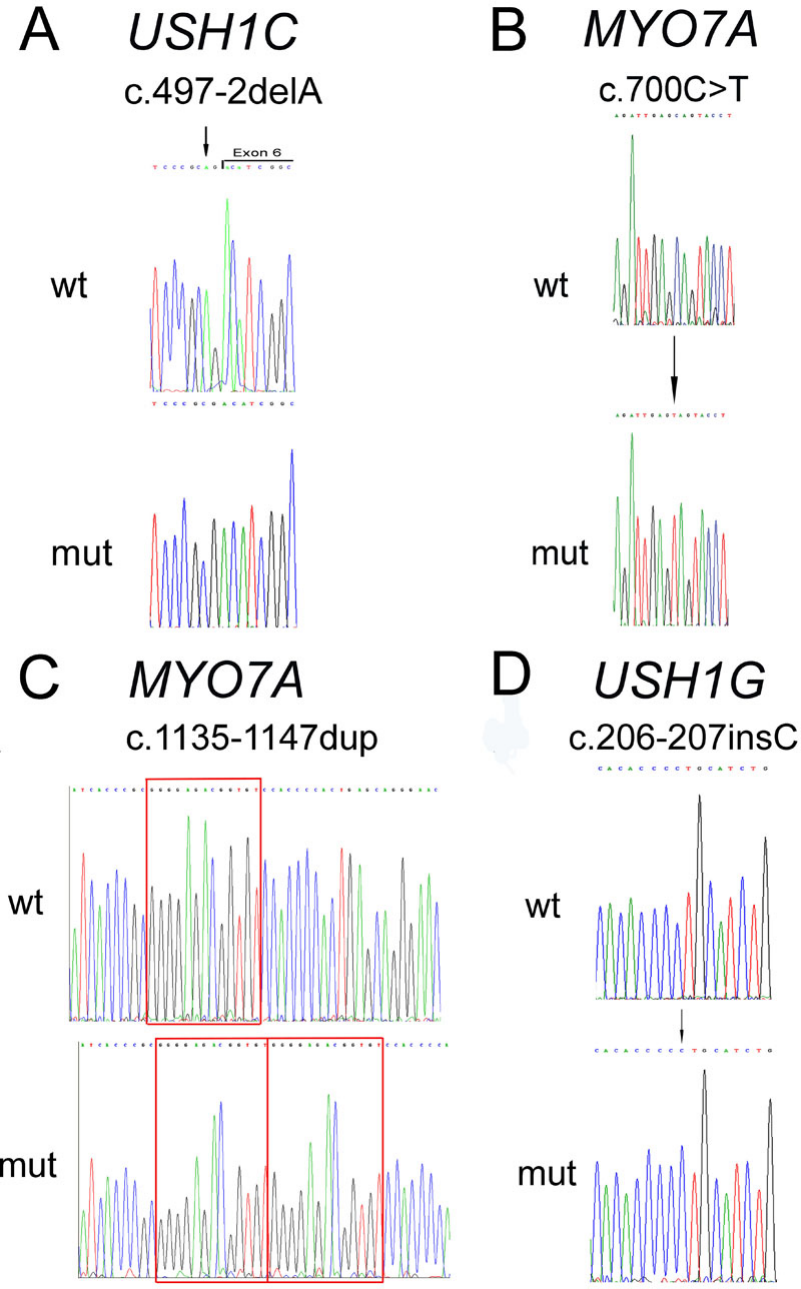
*USH1C*, *MYO7A*, and *USH1G* mutations identified in Israeli Arab families. For each mutation, sequence chromatograms are shown for a non-carrier individual (wt) and an affected individual homozygous for the mutant allele (mut). **A**: The c.497–2delA mutation of *USH1C*. The exon-intron boundary is marked. The deleted base is marked with an arrow on the wt trace. **B**: The c.700C>T (p.Q234X) mutation of *MYO7A.* The mutant base is marked with an arrow. **C**: The c.1135–1147dup mutation of *MYO7A*. The duplicated sequence is marked with a box. **D**: The c.206–207insC mutation of *USH1G*. The inserted base is marked with an arrow.

Haplotype analysis with markers linked to all known USH1 loci indicated co-segregation of a haplotype of two polymorphic marker alleles (D11S902 and D11S4138) linked to the *USH1C* locus on chromosome 11p15.1 with USH1 in this family (Figure 1A). Sequence analysis of the 27 coding exons of the *USH1C* gene, including exon-intron boundaries, revealed a deletion of an A nucleotide at position −2 of intron 5, within the conserved acceptor splice-site (c.497–2delA) (Figure 2A). Analysis of all available family members showed that the mutation co-segregated with USH1 in this family: Affected individuals were homozygotes for the mutation, while their parents were heterozygotes (Figure 1A). The c.497–2delA mutation was previously reported as IVS5–2delA in two unrelated families from Lebanon [9].

### A *MYO7A* mutation in family TB114

Family TB114 is a Muslim Arab family from the Jerusalem area. The parents are first cousins, and two of their three offspring, who were seen by us at the ages of 8 years and 8 months, have bilateral profound SNHL, which was detected at infancy. The affected daughter received a cochlear implant in one ear with very good results. She was recently diagnosed with RP. The affected son is a young baby, and RP has not been diagnosed yet (Figure 1B and Table 2).

Haplotype analysis with markers linked to all known USH1 loci was only partially informative, and the results were non-conclusive. We therefore performed genome-wide homozygosity mapping using the Affymetrix GeneChip Human Mapping 6.0 SNP array. A shared homozygous interval of 57 Mb, composed of 15,470 consecutive SNPs on chromosome 11p13-q14.3, was identified in both affected individuals. This interval contains the gene underlying the USH1B locus, *MYO7A*. Sequence analysis of the 49 coding exons of *MYO7A*, including exon-intron boundaries, was performed in the affected daughter. We identified a homozygous C to T transition that generates a premature stop codon, located in exon 7 (c.700C>T; p.Q234X) (Figure 2B). Analysis of all available family members revealed that the mutation co-segregated with the disease in this family: Affected individuals were homozygotes for the mutation, while their parents and their unaffected brother were heterozygotes (Figure 1B). The p.Q234X mutation has been reported previously in compound heterozygosity with a second *MYO7A* mutation (c.3750+2T>A) in an USH1 patient of unknown ethnicity [10].

### A novel *MYO7A* mutation in family TB63

Family TB63 is a Bedouin family from northern Israel. The parents are first cousins, and two of their eight offspring (Figure 1C, individuals II-1 and II-3) have bilateral profound SNHL, which was detected at infancy. The affected daughter received a cochlear implant at the age of 2. Both were diagnosed with RP at the age of 9 (Table 2).

Haplotype analysis with markers linked to all known USH1 loci indicated co-segregation of a haplotype of four polymorphic marker alleles (D11S4179, D11S1789, D11S4079, and D11S937) linked to the *USH1B* locus on chromosome 11q13.5 with USH1 in this family (Figure 1C). Sequence analysis of the 49 coding exons of the *MYO7A* gene, including exon-intron boundaries, was performed in the affected son. We identified a homozygous 13 bp duplication in exon 11 (c.1135–1147dup; Figure 2C). This novel mutation is expected to lead to a frameshift and premature termination of translation after insertion of 63 irrelevant amino acids, starting at position 383 of the myosin VIIA protein (p.S383W*fs*X63). Analysis of all available family members revealed that the mutation co-segregated with USH1 in this family: Affected individuals were homozygotes for the mutation, while their parents and two of their unaffected siblings were heterozygotes (Figure 1C). The c.1135–1147dup mutation was not identified among 100 chromosomes derived from ethnically-matched normal controls.

Five years after we first saw the family, the affected son married a healthy woman, with normal vision and hearing (Figure 1C, individuals II-1 and II-2, respectively). The two were not related to each other; nevertheless, since they were from the same village, we tested the wife for the c.1135–1147dup mutation, which was identified in her husband. She was found to carry two normal copies of *MYO7A* exon 11. A year later, the couple had a son (Figure 1C, individual III-1). He was diagnosed shortly after birth with severe SNHL. Since he is a young baby, his visual function has not been evaluated yet. Sequencing of *MYO7A* exon 11 revealed that, as expected, he was heterozygote for the c.1135–1147dup mutation. We hypothesized that he may be a compound heterozygote for c.1135–1147dup and a second *MYO7A* mutation, which was either inherited from his mother or generated de novo. We therefore sequenced all coding exons of *MYO7A* in this individual, and found several known polymorphisms but no additional mutations. Sequencing of the single coding exon of *GJB2* (encoding connexin 26), the most common cause for hereditary hearing loss worldwide [11], revealed no mutations either.

### A novel *USH1G* mutation in family TB109

**Figure 3:**
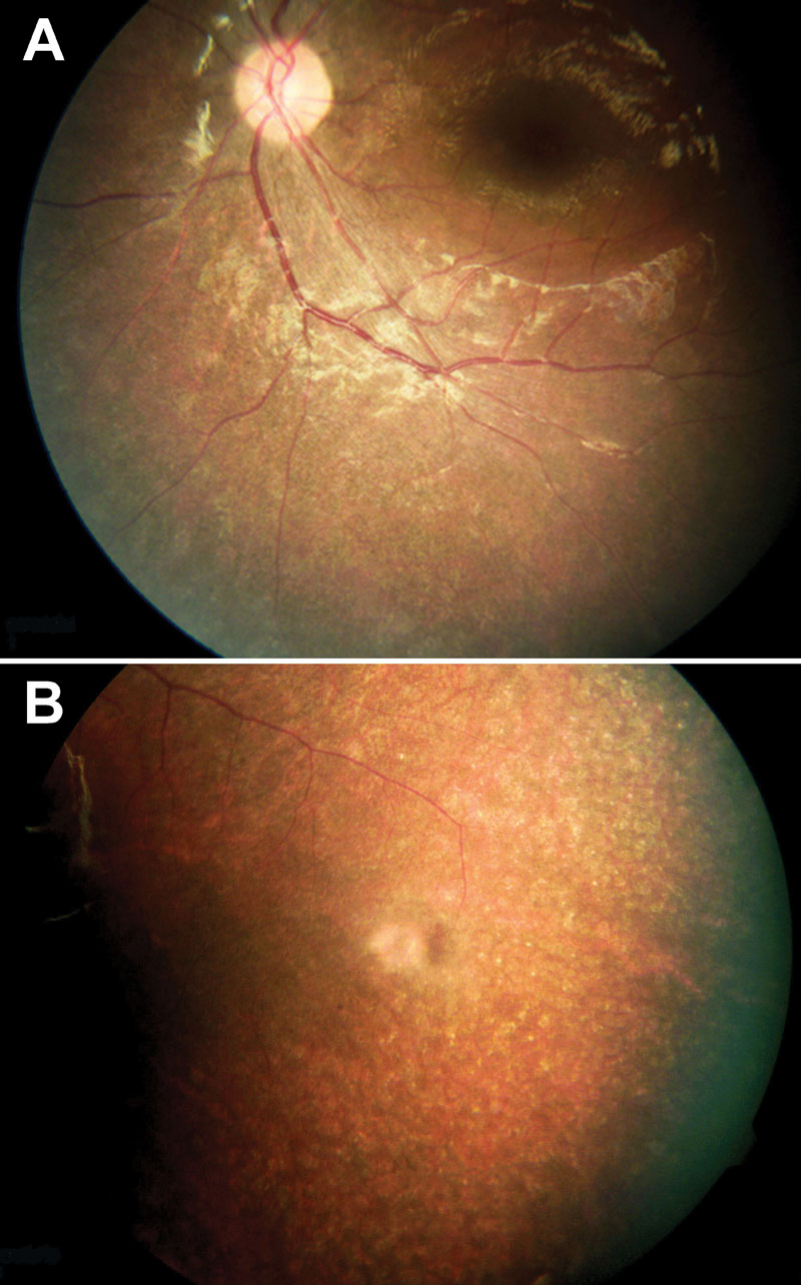
Fundus photographs of affected individuals from family TB109. Fundus photographs of individual II-1 at the age of 8 (**A**), and individual II-2 at the age of 6 (**B**), demonstrating extensive retinal degeneration, salt and pepper–like, retinal pigment epithelial atrophy, reduced foveal reflex, irregularity of vitreoretinal interface, mild vascular attenuation, and absence of peripheral bone spicules or pigment deposits. The optic disc looks normal with no definitive pallor or signs of atrophy.

Family TB109 (Figure 1D) is a Bedouin family from northern Israel. The parents are first cousins, and their two sons have bilateral profound SNHL, which was detected at infancy. Both sons received cochlear implants. They have developmental delay, balance problems, and behavioral problems. RP was diagnosed when the sons were ages of 3 and 5. When we saw the sons, at the ages of 8 and 6, both had reduced visual acuity (BE: 6/10 and BE: 6/12, respectively). Funduscopic examination revealed extensive salt and pepper–like degeneration with mild vascular attenuation, a reduced foveal reflex, and normal optic discs. There were no bone spicules or pigment deposits, although these may appear by the end of the second decade of life (Figure 3). Macular optical coherence tomography was normal (data not shown; Table 2).

Haplotype analysis with markers linked to all known USH1 loci was only partially informative, and the results were non-conclusive. We therefore performed genome-wide homozygosity mapping using the Affymetrix GeneChip Human Mapping 6.0 SNP array. A shared homozygous interval of 7 Mb, composed of 2,224 consecutive SNPs on chromosome 17q25.1, was identified in both affected individuals. This interval contains the *USH1G* gene. Sequence analysis of the three coding exons of *USH1G*, including exon-intron boundaries, was performed in one affected son. We identified a homozygous insertion of a C nucleotide between positions 206 and 207 of *USH1G* cDNA, located in exon 2 (c.206–207insC; Figure 2D). This novel mutation is expected to lead to a frameshift and premature termination of translation after insertion of 66 irrelevant amino acids, starting at position 69 of the SANS protein (p.L69P*fs*X66). Analysis of other available family members revealed that the mutation co-segregated with USH1 in this family: Both affected individuals were homozygotes for the mutation, and their parents are heterozygotes (Figure 1D). The c.206–207insC mutation was not identified among 98 chromosomes derived from ethnically-matched normal controls.

## Discussion

In this study, we identified USH1-causing mutations in four Israeli Muslim Arab families. Of the four mutations identified in these families, two have been reported before. One, the c.497–2delA mutation of *USH1C*, found in family TB86, was previously identified in affected members of two Lebanese families [9]. Based on the common ethnicity (Arab) and relative geographic proximity (Lebanon and northern Israel), the three families may share a common ancestry. The p.Q234X mutation of *MYO7A*, found in family TB114, has been reported previously in a USH1 patient of unknown ethnicity [10,12]. This individual may be related to family TB114; alternatively, p.Q234X may be a recurrent mutation.

In family TB109, we identified a novel *USH1G* mutation (c.206–207insC), which brings the total number of *USH1G* mutations reported to date to eight. Six of these have been associated with a typical USH1 phenotype, including congenital profound SNHL and prepubertal onset of RP. They include five truncating mutations (c.829–848del, c.393insG, c.186–187delCA, p.W38X, and c.206–207insC) and one missense mutation (p.L48P) that appeared in compound heterozygosity with c.186–187delCA ([13,14] and current report). Notably, two novel mutations of *USH1G* were recently reported in individuals with atypical Usher syndrome (p.D458V and c.163_164+13del15). Homozygotes for each mutation had congenital moderate to profound SNHL, normal vestibular function, and mild RP. One is a missense mutation (p.D458V) involving the PDZ binding motif of the SANS protein, and is predicted to reduce the strength of its interaction with harmonin, the protein encoded by the *USH1C* gene [15]. Interestingly, the other one (c.163_164+13del15A) involves nucleotides in the first exon and the first intron of the *USH1G* gene, and is expected to lead to aberrant splicing and generation of a truncated protein [16]. These findings indicate that missense and truncating mutations of *USH1G* can result in a milder USH phenotype, and that the severity of the phenotype induced by *USH1G* mutations may be modified by unknown genetic or environmental factors [16].

Of the ten patients described here, eight are deaf and have RP. The two patients who are only deaf are both young babies, and therefore their retinal function has not been evaluated yet. In USH1, deafness is congenital while the onset of RP occurs within the first decade of life. Individual II-3 in family TB114 is homozygous for the same *MYO7A* nonsense mutation as his older sister, who is deaf and has RP, and he will probably develop RP in the future. As for individual III-1 in family TB63, the situation is more complex. This baby is heterozygous for the *MYO7A* mutation that causes USH1 in his homozygous father and aunt, but we were not able to identify a second *MYO7A* mutant allele. Several explanations are possible for deafness in this child: he may carry a second *MYO7A* mutation located within an intron or in a regulatory region, which therefore we failed to detect; alternatively, his deafness may be due to mutation(s) in a different gene (independent segregation of mutations in more than one deafness-causing gene within the same family has been previously reported [4]). A third possibility is that deafness in this individual is due to a non-genetic etiology, as genetics accounts for only about 50% of congenital deafness cases [17]. Whether this individual develops RP depends on the nature of his deafness (genetic versus environmentally-caused), and of the additional mutation(s) he carries (if at all), and cannot be predicted at this stage.

USH1 is a genetically heterogenous condition. Seven USH1 loci have been mapped, and the causative genes have been identified for five [1]. As of September 2011, 655 different mutations for USH1 are listed in the UMD-USHbases, a comprehensive set of databases that records pathogenic mutations and unclassified variants in seven USH-causing genes [18]. Of these, 52% are in the *MYO7A* gene, followed by *CDH23* (28%), *PCDH15* (13%), *USH1C* (6%), and *USH1G* (1%). As indicated by these numbers, *USH1C* and *USH1G* are the rarest contributors to USH1 etiology worldwide. Mutations of *USH1C* are a rare cause of USH1, except in the Acadian population, which segregates a founder mutation in this gene [13,19,20]. For *USH1G*, only seven mutations have been reported to date [13–16]. It is therefore interesting that two of the four Israeli Arab families reported here have mutations in these two genes. This finding further demonstrates the unique genetic structure of the Israeli population in general, and the Israeli Arab population in particular, which due to high rates of consanguinity segregates many rare autosomal recessive genetic conditions [6].
